# HIGH RUMINATION AND LOW AMINO ACIDS ARE ASSOCIATED WITH AN INCREASED RISK OF METABOLIC SYNDROME

**DOI:** 10.1093/geroni/igz038.3466

**Published:** 2019-11-08

**Authors:** Jessie Alwerdt, Yuan Tian, Andrew D Patterson, Martin Sliwinski

**Affiliations:** 1 Penn State University, Center for Healthy Aging, University Park, Pennsylvania, United States; 2 Penn State University, Huck Institutes of the Life Sciences, University Park, Pennsylvania, United States; 3 Penn State University, The Department of Veterinary and Biomedical Sciences, University Park, Pennsylvania, United States

## Abstract

Metabolic syndrome (MetS) is an increasing epidemic worldwide. Identifying modifiable behaviors that intersect with the association between MetS and associated metabolites could result in alternative methods to prevent those at risk for MetS. Here we investigate the moderation of ruminating thought processes between metabolites and MetS. Rumination has been linked to exacerbating the physiological response of stress and increasing the risk for poor health outcomes, such as hypertension. Data consisted of 180 middle-aged adults from Bronx, NY. MetS was calculated based on the NIH guidelines using waist, triglycerides, high-density lipoproteins, blood pressure, and glucose. Using NMR-based metabolomics, 26 serum metabolites were obtained. The Rumination-Reflection Questionnaire measured rumination of thoughts. Interactions between rumination and each metabolite were conducted with logistic regressions (e.g., Valinexrumination). Overall, significant moderation occurred with the greatest effect involving different levels of phenylalanine, betaine, creatine, and isoleucine with higher rumination in relation to the increased probability for MetS. The greatest risk for MetS was in those who were high ruminators and had low values of these AAs. Therefore, within those who are high ruminators, an increase in these AAs may be beneficial in improving the risk for MetS. Further, in those who are low ruminators, minimal moderation occurred. AAs disturbance has been linked with MetS in past studies, as well as in mental health. These results suggest that ways of handling thoughts that are intertwined with everyday stress may exacerbate these associations and could benefit with modification.

